# Efficient Heat Dissipation of Uncooled 400-Gbps (16×25-Gbps) Optical Transceiver Employing Multimode VCSEL and PD Arrays

**DOI:** 10.1038/srep46608

**Published:** 2017-04-18

**Authors:** Tien-Tsorng Shih, Yu-Chieh Chi, Ruei-Nian Wang, Chao-Hsin Wu, Jian-Jang Huang, Jau-Ji Jou, Tai-Cheng Lee, Hao-Chung Kuo, Gong-Ru Lin, Wood-Hi Cheng

**Affiliations:** 1 Department of Electronic Engineering, National Kaohsiung University of Applied Sciences, Kaohsiung 80778, Taiwan; 2 Graduate Institute of Photonics and Optoelectronics, National Taiwan University, Taipei 10617, Taiwan; 3 Department of Electrical Engineering, National Taiwan University, Taipei 10617, Taiwan; 4 Department of Photonics, National Chiao Tung University, Hsinchu 30050, Taiwan; 5 Graduate Institute of Optoelectronic Engineering, National Chung Hsing University, Taichung 40227, Taiwan

## Abstract

An effective heat dissipation of uncooled 400-Gbps (16×25-Gbps) form-factor pluggable (CDFP) optical transceiver module employing chip-on-board multimode 25-Gbps vertical-surface-emitting-laser (VCSEL) and 25-Gbps photodiode (PD) arrays mounted on a brass metal core embedded within a printed circuit board (PCB) is proposed and demonstrated. This new scheme of the hollow PCB filling with thermally-dissipated brass metal core was simulated and used for high temperature and long term stability operation of the proposed 400-Gbps CDFP transceiver. During one-hour testing, a red-shift of central wavelength by 0.4-nm corresponding temperature increment of 6.7 °C was observed with the brass core assisted cooler module. Such a temperature change was significantly lower than that of 28.3 °C for the optical transceiver driven with conventional circuit board. After 100-m distance transmission over a multimode fiber (OM4), the 400-Gbps CDFP transceiver exhibited dispersion penalty of 2.6-dB, power budget of ≧ 3-dB, link loss of ≦ 0.63-dB, mask margin of 20%, and bit error rate (BER) of <10^−12^ with maintained stability more than one hour. The developed 400-Gbps CDFP transceiver module employing low-power consumption VCSEL and PD arrays, effective coupling lens arrays, and well thermal-dissipation brass metal core is suitable for use in the low-cost and high-performance data center applications.

The explosive development of internet technology has urged the rapidly increased data streaming capacity requirement from live webcast, personal cloud data up/down loading, clients-to-servers transmission, and high-definition multimedia. Especially, the data centers are massively constructed because of the rapidly grown data traffic from cloud computing and mobile networks. To reduce the traffic time and improve the transmission quality of the data stream, the optical transceiver persistently advanced its maximal allowable data rate over past decade^1^,^2^,^3^,^4^,^5^,^6^,^7^,^8^,^9^,^10^,^11^,^12^,^13^,^14^,^15^,^16^. For rapid evolution of data center, the specification of optical transceivers has been updated to 400 Gbps^17^,^18^,^19^,^20^, and the multimode vertical-cavity-surface-emitting-lasers (VCSELs) at 850 nm with compact structure and high modulation bandwidth have emerged for practical applications^21^,^22^,^23^,^24^,^25^,^26^,^27^,^28^,^29^,^30^,^31^,^32^,^33^,^34^,^35^. The 850-nm multimode VCSELs with intriguing features including cost-effectiveness and low-power consumption, which have been proven to be a reliable key component in low-cost multimode fiber based optic interconnects for data center applications^36^.

Remarkably, Peters *et al*. developed an 850-nm VCSEL for 10-Gbps operation at high temperatures in 2001^21^. By directly modulating an oxide-confined 850-nm VCSEL, the error-free transmission at 35-Gbps over 100-m-long multimode fiber was demonstrated by Westbergh *et al*. in ref. 24. Later on, the same group promoted the error-free data transmission up to 44-Gbps^28^. In 2014, Moser *et al*. designed an oxide-confined 980-nm VCSEL for very-short-reach optical interconnects, which achieved 46-Gbps error-free data transmission at 85 °C^25^. Kuchta *et al*. demonstrated a directly modulated 850-nm VCSEL based optical link achieving 64-Gbps over 57-m in OM4 fiber^30^. In 2015, the same group employed an equalizer incorporated driver/receiver circuit to perform 50-Gbps transmission with VCSEL at 30–90 °C^27^. Furthermore, Szczerba *et al*. used a similar device to implement 56-Gbps 8-pulse-amplitude-modulation (PAM) transmission over 50-m-long multimode fiber (MMF)^31^. The diving IC and feed-forward equalization enabled the 850-nm multimode VCSEL for 71-Gbps on-off-keying (OOK) transmission^35^, and the 54-Gbps link over 2.2-km MMF had successfully approached^36^.

For data-center application, the integrated VCSEL array could be operated for >100-Gbps applications^37^,^38^,^39^,^40^,^41^,^42^,^43^,^44^,^45^,^46^,^47^,^48^. Parallel interconnects of 25-Gbps multimode VCSELs in combination with parallel ribbon multimode fiber have offered a clear path on connecting the data center networks at up to 400-Gbps or more data rate transmission potentially. In early 2004, Kuchta *et al*. employed a GaAs VCSEL array to propose a 120-Gbps optical link through a 316-m-long MMF channel^38^. In 2008, Schow *et al*. used a 24-channel 850-nm optical transceiver with CMOS-based driver to achieve a 300-Gbps aggregated bi-directional data rate^39^. In 2011, the same group employed polymer waveguide integrated printed circuit board (PCB) to demonstrate a 225-Gbps bidirectional optical link with 15 channels at 15-Gbps^40^. In 2013, Ghiasi *et al*. investigated the feasibility and limitation of 850-nm VCSELs based 100-GbE applications with 4 × 26-GBd signaling, in which the traditional feed-forward and decision-feedback equalizers were used to overcome optical and electrical channel impairments^43^. In 2015, Westbergh *et al*. proposed 2-D six-channel 850-nm VCSEL arrays to achieve 40-Gbps error-free transmission at 85 °C, which enabled an aggregate capacity of 240-Gbps over a single multicore fiber^42^. A 28-Gbps × 24-channel clock-and-data recovery (CDR) integrated VCSEL optical transceiver module with a data rate density of 1344 Gbps/inch^2^ operated under relatively low link power was also proposed by Nagashima *et al*.^45^. In addition, Tsunoda *et al*. also developed a high-density multi-rate VCSEL transceiver with CDRs to suppress the jitter of optical link for approaching a 24 to 34-Gbps × 4 interconnect^46^. However, the operating environment for the optical components in data center may possibly reach high temperature without cooling package, as the density of bandwidth and parallel interconnect amount of the optical components and driver chips increase dramatically. Furthermore, the serial speed of the optical links is also increased by at least of a factor of 2 with each new generation. Therefore, the demonstration of optical links error-free running at temperatures beyond the expected operating range is one of the important steps toward commercial viability in data center applications. Despite numerous studies of thermal management in data center applications^49^,^50^, limited information is available on thermal management for optical components, such as optical transceiver module used in data center. In conventional optical transceiver modules, the thermal managements have been comprehensively investigated for optimizing the cooling and thermal dissipation of opto-electronic device package. Chen *et al*.^51^, Liu *et al*.^52^ and Mutig *et al*.^53^ reported device-level investigations on the thermal characteristics of the VCSEL. The thermal resistance of an integrated VCSEL array on PCB was studied by Lee *et al*.^54^, Pu *et al*.^55^, Krishnamoorthy *et al*.^56^, and Choi *et al*.^57^. Later on, versatile cooling and heat dissipation schemes within the module package were represent by Hino *et al*.^58^, Brusberg *et al*.^59^, Romero *et al*.^60^, Doany *et al*.^61^, and Uemura *et al*.^62^. Except the thermal stabilization on the VCSEL packed in the optical transceiver modules, the laser diode driver, the trans-impedance amplifier, and the CDR circuit chips are also the heat sources in addition to opto-electronic devices. In view of previous solutions, it is easily observed that a smart design with compact and high-efficient cooling capability is mandatory for the 400 G transceiver PCB with dense power devices. When considering the efficient thermal dissipation, a brass block embedded within the PCB of module for mounting both the circuit chips and opto-electronic devices can serve as a superior heatsink applicable to commercial optical transceivers for improving transmission performance.

In this study, we propose a novel scheme of thermoelectrically separated PCB with superior heat dissipation ability to maintain the temperature stability of the VCSEL/PD and its driver circuit for demonstrating an uncooled error-free 400-Gbps (16×25-Gbps) form-factor pluggable (CDFP) optical transceiver module. To deal with the thermal dissipation for long-term stability during operation, the PCB under the driver IC is excavated to fill up a brass block for thermoelectrically separated heat dissipation of the 400-Gbps CDFP transceiver, which significantly cools down the driver ICs and nearby PCB with a compact thermoelectric separation brass mesa. The numerical simulation on the temperature distribution contour with or without the brass block based thermoelectric separator is demonstrated to present the cooling performance of the 400-Gbps CDFP optical transceiver module. This excellent thermal dissipation with brass block embedded in the PCB of the proposed 400-Gbps transceiver module is experimentally performed even under high temperature and long-term operation. The design principle and analytic results of the 400-Gbps CDFP transceiver module are discussed in detail to support the transmitted data quality at back-to-back (BTB) and 100-m OM4 multimode fiber (MMF) transmission cases. Such unique design of the thermoelectrically separated 400-Gbps CDFP optical transceiver reveals an ultra-stable heat dissipation at relatively low temperature with uncooled PCB design to provide a crosstalk-free optical link for intra-data-center applications.

## Results

**Figure 1:**
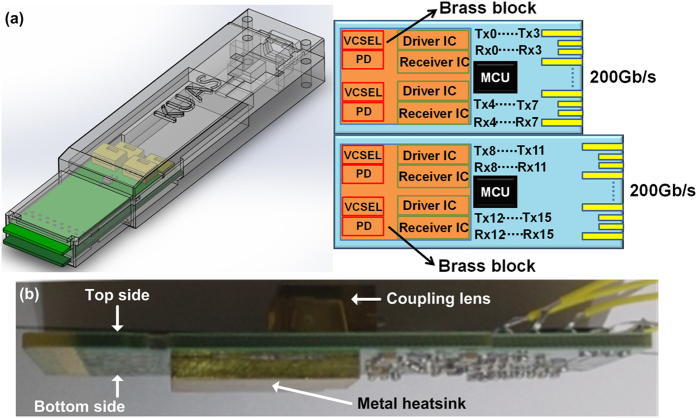
Heat dissipation of a 400-Gbps CDFP optical transceiver module. (**a**) The design of the 400-Gbps CDFP optical transceiver module; (**b**) The image of a 100-Gbps transceiver with brass block based metal heatsink.

**Figure 2:**
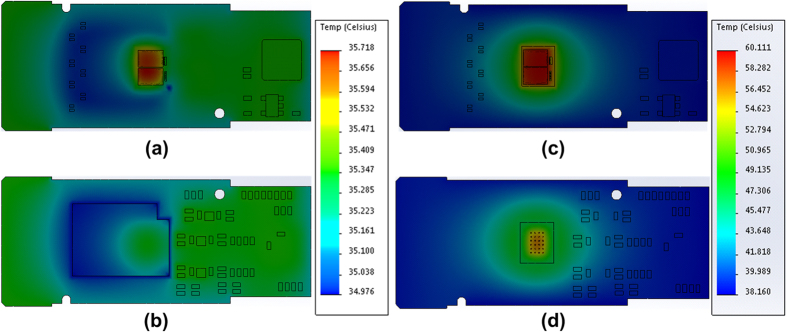
The simulated temperature distributions of the 100-Gbps optical transceiver with and without brass block based metal heatsink. Simulated temperature distributions of the thermoelectrically separated 100-Gbps transceiver at (**a**) top and (**b**) bottom PCB surfaces; Simulated temperature distributions of the 100-Gbps transceiver at (**c**) top and (**d**) bottom PCB surfaces for the case without using the brass block based metal heatsink.

In experiment, the employed 400-Gbps CDFP transceiver module consists of 16-channel VCSEL transmitters and p-i-n PD receivers (on both upper and lower PCBs), in which all channels enable 28-Gbps data rate to provide total data rate of 400-Gbps, as shown in Fig. 1(a). To verify the ability of the proposed thermoelectric separation structure, a conventional 100-Gbps transceiver with single PCB design is employed with its driver IC chip bond region excavated to fill up the brass block, as shown in Fig. 1(b). When the operating bias current of the VCSEL is set at 7 mA, the top- and bottom-side temperature distributions of the 100-Gbps optical transceiver with and without thermoelectric separation are simulated by using the SolidWorks software, as shown in Fig. 2. During simulation on temperature distribution, the thermal effect of micro-control unit (MCU) and other components on the driving circuit are ignored, as the temperature variation of whole PCB is mainly predominated by the driver IC chip. With the aid of brass block based metal heatsink, the 100-Gbps optical transceiver is able to stabilize its temperature at <35.7 °C, which is significantly lower than that at <60.1 °C for the case without the brass block based metal heatsink. Since the metal heatsink is placed at the bottom side of the 100-Gbps transceiver, the bottom PCB surface temperature is relatively lower than the top PCB surface. The temperature differences between top and bottom PCB surfaces for the 100-Gbps transceiver with and without thermoelectric separation are <0.3 °C (T_top_: 35.7 °C and T_bottom_: 35.4 °C) and <3.4 °C (T_top_: 60 °C and T_bottom_: 56.6 °C), respectively. When considering a typical operating power of 1.6 W for the 100-Gbps transceiver, the corresponding thermal resistances of 0.19 °C/W and 2.1 °C/W are observed, respectively for the case with and without thermoelectric separation, and a distinct reduction of the thermal resistance by more than one order of magnitude can be obtained with the brass block aided heatsink.

**Figure 3:**
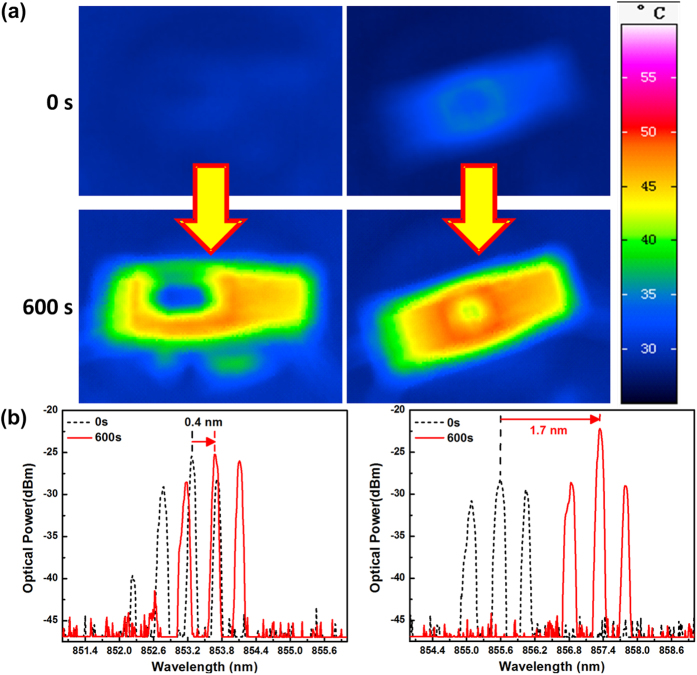
Experimental measurements of the 100-Gbps optical transceiver with and without brass block based metal heatsink. (**a**) The PCB bottom surface thermal images of the 100-Gbps optical transceiver with (left) and without (right) heat dissipation; (**b**) The VCSEL emitting spectra of the 100-Gbps optical transceiver with (left) and without (right) thermoelectric separation for one hour testing.

As a result, the measured top- and bottom-side thermal images of the 100-Gbps optical transceiver without and with thermoelectric separation is shown in Fig. 3(a), indicating that the bottom PCB surface temperatures without and with the brass block based metal heatsink are 47 °C and 37 °C, respectively. In addition, the measured temperature of surrounding areas is higher than that of intermediate region when all components on the driving circuit are under normal operation. Obviously, the use of with brass block based metal heatsink has already revealed better temperature stabilization than the case without embedded brass block. This result shows a good agreement with the numerical simulation. The significant deviation on the obtained optical spectra of the 100-Gbps transceiver with and without thermoelectric separation is shown in Fig. 3(b). Note that the temperature-stabilized 100-Gbps transceiver only red-shifts its central wavelength by 0.4-nm after one hour. When considering that the temperature dependent wavelength red-shift is 0.06 nm/°C for a typical VCSEL, such a wavelength red-shift corresponds to a related temperature change of 6.7 °C during one-hour operation. For comparison, a significantly enlarged wavelength red-shift of up to 1.7-nm is observed for the case without using the brass block based metal heatsink, corresponding to a temperature change as high as 28.3 °C and verifying that the metal heatsink is mandatory to achieve the efficient heat dissipation and long-term temperature stabilization for the 100-Gbps optical transceiver.

**Figure 4:**
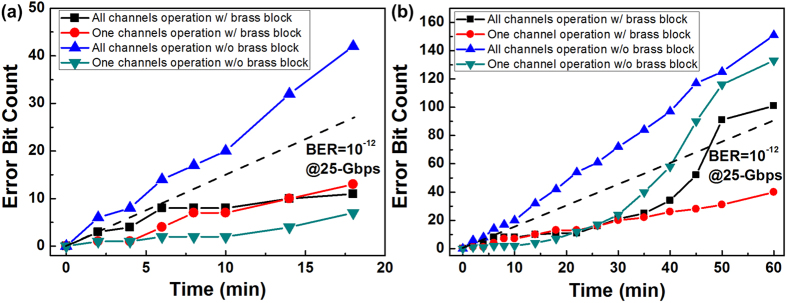
Error bit counts measurements of the 100-Gbps optical transceiver with and without brass block based metal heatsink. (**a**) Error bit counts within 18 minutes for the optical transceiver with and without brass block; (**b**) Error bit counts within an hour for the optical transceiver with and without brass block.

For the optical transceiver with and without brass block, the error bits after BTB transmissions were counted continuously within 18 min and 60 min, as respectively shown in Fig. 4. According to the VCSEL spectra analyses, the wavelength shift and the temperature of transceiver can be stabilized after operation for 3 minutes. Therefore, the counted error bits for the optical transceiver with and without brass block after BTB transmissions were persistently monitored and compared within 18 minutes, as shown in Fig. 4(a). For a single channel turned on without and with brass block in the optical transceiver circuit board, the bit error rates (BERs) were determined as 4.81 × 10^−13^ and 2.59 × 10^−13^, respectively. When turning on all channels, the optical transceiver without brass block reveals a BER of 1.56 × 10^−12^, whereas the optical transceiver with brass block reduces its BER to 4.07 × 10^−13^. With the use of the embedded brass block based metal heatsink, all channels of the transceivers with brass block can operate below the BER criterion of 1 × 10^−12^ to confirm the negligible influence of thermal crosstalk on the core temperature of transceivers in between. By lengthening the test duration up to 60 min, all channels without brass block remain almost fixed error bit count slope with a BER of 1.67 × 10^−12^ after 60-min operation, as shown in Fig. 4(b). With the assistance of the brass block, the error bit slope count only shows slightly rising trend after 35-min operation due to gradually exhausted heatsink capability of the brass block. Even though, the highest BER still maintains below 6.67 × 10^−13^ after 60-min operation. In comparison with a single-channel case, the optical transceiver without or with brass block exhibits relatively low BER of 1.2 × 10^−12^ and 4 × 10^−13^ with same duration of operation. These results confirm our heatsink design with embedded brass block can rapidly and persistently stabilize the temperature of the transceiver to perform improved BER response.

In fact, the temperature of transceiver can be stabilized just after 3 minutes according to the VCSEL spectra measurements. The reason of the increased error bit counts after 30 minutes could be not only attributed to the heatsink capacity. During long-term test, the transceiver module was not set up on a vibration-free optical table. Besides, the bare transceiver module does not have outer housing and LC adapter, so the ribbon fiber cable could not be tightly plugged in the transceiver module without cable locking mechanism. In addition, the connector of the ribbon fiber was not tightly plugged into lens array. As testing time lengthens, the connector could be gradually sagged and the module could not always connect stably with the ribbon fiber cable. Owing to these reasons, the transmission light power could vibrate and the error bits could increase unexpectedly. Even though, the very low BER can still be obtained within one-hour operation.

**Figure 5:**
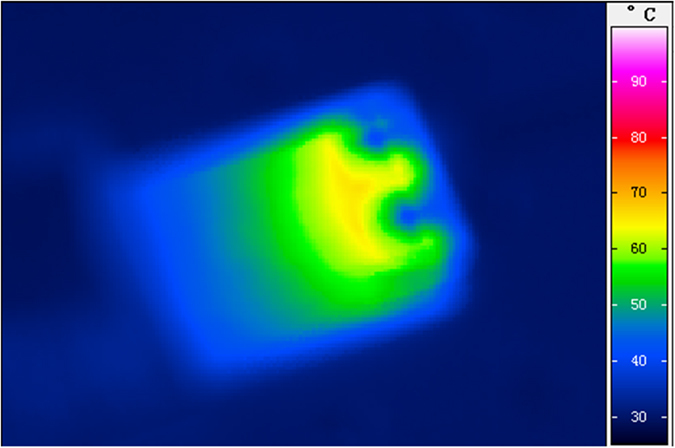
The PCB bottom surface thermal image of the 400-Gbps CDFP optical transceiver.

Furthermore, the same thermoelectric separation structure is implemented to cool down and stabilize the PCB temperature on the driver IC part of the 400-Gbps CDFP transceiver, and the corresponding thermal image is shown in Fig. 5 to demonstrate a good thermal dissipation and stabilization with the aid of the brass block based metal heatsink. Note that the bottom PCB surface temperature of the 400-Gbps CDFP transceiver efficiently cools down to only 43 °C, as compared to a higher temperature of >70 °C for the same 400-Gbps CDFP transceiver without thermoelectric separation design.

**Figure 6:**
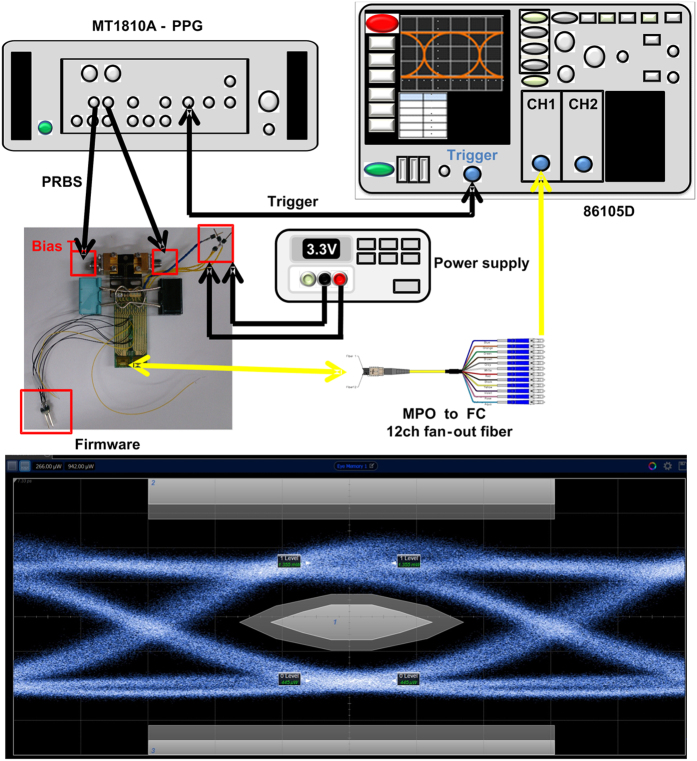
The optical eye-diagram measurement of the thermoelectrically separated 400-Gbps CDFP optical transceiver. (**a**) The experimental setup for measuring the BTB transmitted eye diagram of the thermoelectrically separated 400-Gbps CDFP optical transceiver; (**b**) The measured optical eye diagram of the thermoelectrically separated 400-Gbps CDFP transceiver carried 25-Gbps data with corresponded eye pattern mask.

**Figure 7:**
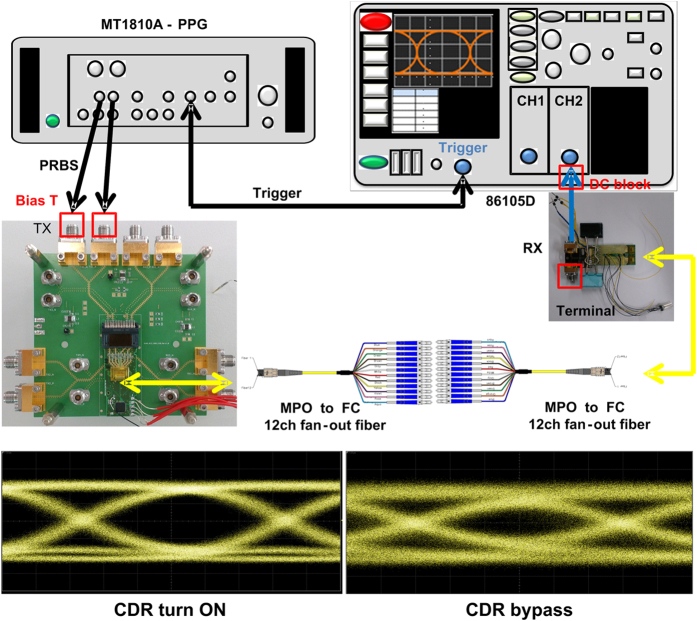
The electrical eye-diagram measurement of the thermoelectrically separated 400-Gbps CDFP receiver. (**a**) The experimental setup of receiver eye diagram measurement; (**b**) The received eye diagrams with and without CDR functionality.

**Figure 8:**
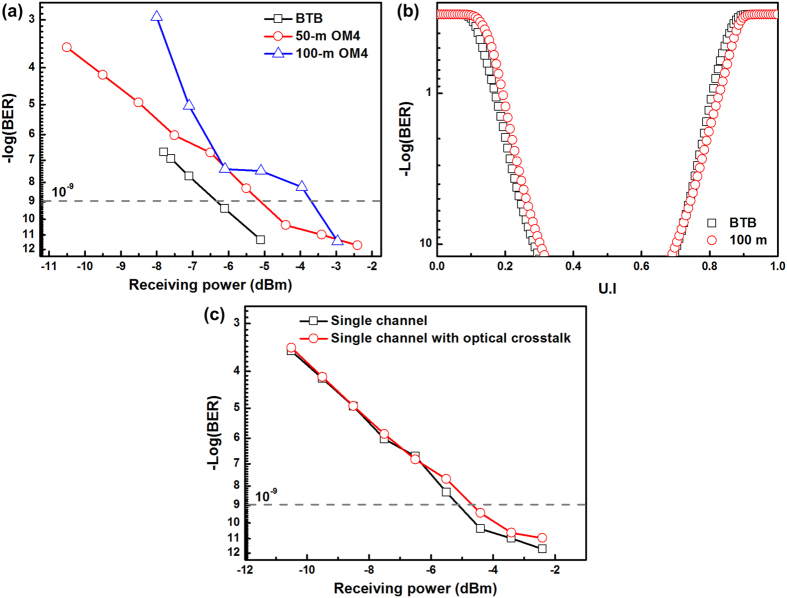
The transmission performances of temperature-stabilized 400-Gbps CDFP optical transceiver. (**a**) The measured BERs after BTB, 50-, and 100-m OM4 MMF transmissions; (**b**) The bathtub curves under BTB and 100-m OM4 MMF transmissions; (**c**) The optical crosstalk measurement.

After thermal cooling temperature stabilization with the brass block, the single-channel transmission performance of the 400-Gbps CDFP transmitter is characterized, as shown in Fig. 6(a). As a result, the Fig. 6(b) shows the measured optical eye-diagram of the received 25-Gbps PRBS data with corresponding eye pattern mask, which exhibits an signal-to-noise ratio (SNR) of 8.3-dB, a rising/falling time of 15.2/18.9 ps, a peak-to-peak timing jitter of 12.8 ps, an extinction ratio (ER) of 4.8-B, and a mask margin of 25.6%. Moreover, the experimental setup for testing the single-channel receiver performance is shown in Fig. 7(a). Due to an optimized wire bonding configuration, the RF performance in this design is similar to the original without brass block design. During operation, the CDR function on channel of the CDFP transceiver always turns on, which helps to obtain a clear eye-diagram with an SNR of 9-dB, a rising/falling time of 17.8/17.4 ps and a peak-to-peak timing jitter of 13.2 ps, as shown in Fig. 7(b). When bypassing the CDR functionality, the eye-diagram blurs with a decreased SNR of 5.2-dB, a lengthened rising/falling time of 19.8/28.5 ps, and an enlarged peak-to-peak timing jitter of 30.4 ps. To lengthen the transmission distance for intra-data center application, the BER responses of the heat dissipated 400-Gbps CDFP transceiver after propagating along OM4 MMF with different distances are shown in Fig. 8(a). To meet the data communication required BER of 10^−9^, a receiving power sensitivity of −6.4-dBm is obtained for the BTB case. After OM4 MMF transmission with distance lengthening from 50 to 100-m, the receiving power sensitivities are slightly increased from −5.1 to −3.8-dBm with corresponding power penalty enlarged from 1.2 to 2.6-dB, when comparing with the BTB case. At a receiving power of −3-dBm, a BER of 3.7 × 10^−12^ is observed for the 100-m-long OM4 MMF case. Furthermore, the Fig. 8(b) compares to the bathtub curves of the temperature-stabilized 400-Gbps CDFP transceiver after BTB and 100-m OM4 MMF transmissions. Note that the similar jitter tolerances of 0.5 U.I. are observed for both cases, indicating that the brass block based metal heatsink essentially guarantees the transmission performance of the 400-Gbps CDFP optical transceiver. Comparing with an original without brass block design, this module maintains a better transmission performance after a continuous one hour operation.

Since the 400-Gbps CDFP optical transceiver has 16 channels totally, all channels are simultaneously turned on for optical crosstalk analysis, as shown in Fig. 8(c). In comparison with the single-channel case, a power penalty of only 0.48-dB is observed at BER of 10^−9^ to guarantee the nearly identical transmission performance for 16 channels of the 400-Gbps CDFP transceiver with its PCB hollowing out and filling up the brass block based metal heatsink.

## Discussion

A new scheme of thermoelectrically separated PCB to fill up a brass block with superior heat dissipation ability to maintain the temperature stability of an uncooled 400-Gbps (16×25-Gbps) CDFP optical transceiver module has been successfully demonstrated. With the aid of proposed brass block based metal heatsink, the 100- and 400-Gbps optical transceivers were able to stabilize their temperatures at <35.7 °C and <43 °C for one-hour testing, respectively. The module temperatures of the 100- and 400-Gbps transceivers were significantly lower than that at <60.1 °C and >70 °C for the cases without the brass block based metal heatsink, respectively. During one-hour testing, the central wavelength red-shifted by 0.4-nm with respect to the temperature increment of 6.7 °C was observed for the optical transceiver with the brass block assisted cooler module. Such a red-shift was far smaller than that observed in conventional PCB board without cooling caused by the temperature drift of up to 28.3 °C. For single-channel 25-Gbps PRBS data transmission, the 400-Gbps CDFP transceiver exhibited a clear eye diagram with mask margin >25%, corresponding to an SNR of 8.3-dB and a peak-to-peak timing jitter of 12.8 ps. The BTB transmission with receiving power sensitivity of −6.4 is obtained. After 100-m OM4 MMF transmission, the 400-Gbps CDFP transceiver module exhibited a power budget of ≧3-dB, a link loss of ≦0.63-dB, a mask margin of 20%, and a receiver BER of <10^−12^ with maintained stability more than one hour. Furthermore, the similar jitter tolerances of 0.5 U.I. were observed for BTB and 100-m OM4 MMF cases. When turning on the 16 optical channels simultaneously, the optical crosstalk penalty was only 0.48-dB. These results indicated that the brass metal core heatsink can essentially stabilize the temperature drift to improve the transmission performance of the 400-Gbps CDFP optical transceiver. Apparently, the brass block based heat dissipation core with excellent temperature cooling and stabilizing abilities essentially enabled the proposed 400-Gbps optical transceiver module for low-cost and high-performance data center applications. The unique design of the brass metal core embedded within PCB to provide superior heat dissipation was effectively cools down the lens-coupled VCSEL/PD array as well as driver circuits to benefit from advantages of low-power consumption and cost-effectiveness of optical components with a highly efficient design on thermal management.

## Methods

### Design and measurement of 400-Gbps CDFP optical transceiver

For packaging the standard 400-Gbps CDFP optical transceiver module, the 16 channels of the VCSEL/PD package with lens array and CDR IC driver were hybrid integrated on the PCB. The 45° lensed fiber array was used for effectively coupling the VCSEL output to the PD through fiber ribbon. In transmitting part, the CDFP transceiver consists of high performance 25-Gbps VCSELs and driver ICs, and a MCU controls the driving circuit to provide bias and modulating currents for directly encoding the VCSEL. The receiving part mainly consists of high bandwidth 25-Gbps photodiodes and receiver ICs. The receiver IC is a multi-functional device which combines the electrical ransimpedance amplifier (TIA), limiting amplifier (LA) and CDR module together. Such a design can reduce timing jitter and noise effectively. To deal with the thermal dissipation of the 400-Gbps CDFP transceiver, the PCB under the driver IC is excavated to fill up a brass block, and the thermal image is experimentally monitored by using a high-resolution thermographic 2-D mapper (InfraTec, VarioCAM^®^ HDx head).

To measure the transmission performance of the 400-Gbps CDFP transceiver, a power supply provides 3.3-V DC voltage for the driver IC to start up the transmission of the VCSEL/PD in the 400-Gbps CDFP transceiver. The positive and negative 25-Gbps PRBS data with a pattern length of 2^23^−1 generated from a commercial pattern generator (PPG, Anritsu MT1810A) was used to differentially encode the VCSEL transmitter. After BTB transmission with a multi-fiber push (MPO) onto ferrule connector (FC) 12-channel fan-out fiber, the optical 25-Gbps data was received and analyzed by the optical module of a digital sampling oscilloscope (DSO, Keysight 86105D). Moreover, for testing the single-channel receiver performance, a standard 100-Gbps (25 × 4-channel) transmitter is employed to deliver an optical 25-Gbps PRBS data. By using a pair of MPO to FC 12-channel fan-out fibers, the optical data was received by the receiving part of the 400-Gbps CDFP optical transceiver. Then, the received data after amplification with the cascaded TIA and LA module was sent into the CDR module and finally analyzed by the electrical module of the DSO.

## Additional Information

**How to cite this article**: Shih, T.-T. *et al*. Efficient Heat Dissipation of Uncooled 400-Gbps (16×25-Gbps) Optical Transceiver Employing Multimode VCSEL and PD Arrays. *Sci. Rep.* **7**, 46608; doi: 10.1038/srep46608 (2017).

**Publisher's note:** Springer Nature remains neutral with regard to jurisdictional claims in published maps and institutional affiliations.

## References

[b1] Tomoyuki H. . A 10Gbps × 12 channel pluggable optical transceiver for high-speed interconnections. 2008 58th Electronic Components and Technology Conference, Lake Buena Vista, FL, USA. doi: 10.1109/ECTC.2008.4550231. (2008, May 27–30).

[b2] Kanazawa S. . A compact EADFB laser array module for a future 100-Gbps Ethernet transceiver. IEEE J. Sel. Top. Quantum Electron. 17, 1191–1197 (2011).

[b3] Yagisawa T. . 200-Gbps compact card-edge optical transceiver utilizing cost-effective FPC-based module for optical interconnect. 2012 38th European Conference and Exhibition on Optical Communications, Amsterdam, Netherlands. doi: 10.1364/ECEOC.2012.We.1.E.3. (2012, Sept. 16–20).

[b4] Sugawara T. . A compact, high-optical-coupling-efficiency transceiver with lens-integrated optical devices and high-capacity 25-Gbps/ch×12-ch flexible waveguides. Optical Fiber Communication Conference, Los Angeles, CA, USA. doi: 10.1364/OFC.2012.OTh1E.2. (2012, Mar. 4–8).

[b5] Chaciński M., Chitica N., Molin S., Lalic N. & Sahlén O. 25.78 Gbps data transmission with 850nm multimode VCSEL packaged in QSFP form factor module. Optical Fiber Communication Conference, Anaheim, CA, USA. doi: 10.1364/OFC.2013.OW1B.1. (2013, Mar. 19–21).

[b6] Matsushima N., Chujo N., Takai T. & Yazaki T. A 25 Gbps × 4-ch, 8 × 8 mm^2^ small size optical transceiver module for optical interconnection. 2013 3rd IEEE CPMT Symposium Japan, Kyoto, Japan. doi: 10.1109/ICSJ.2013.6756089. (2013, Nov. 11–13).

[b7] Doi Y., Ohyama T., Yoshimatsu T., Soma S. & Oguma M. 400GbE demonstration utilizing 100GbE optical sub-assemblies and cyclic arrayed waveguide gratings. Optical Fiber Communication Conference, San Francisco, California, USA. doi: 10.1364/OFC.2014.M2E.2. (2014, Mar. 9–13).

[b8] Alexis C., Stadtler W. & Pez M. 10 Gbps multiple channel optical transceivers for harsh environment applications. 2014 IEEE Avionics, Fiber-Optics and Photonics Technology Conference (AVFOP), Atlanta, GA, USA. doi: 10.1109/AVFOP.2014.6999462. (2014, Nov. 11–13).

[b9] Kanazawa S. . 400-Gbps operation of flip-chip interconnection EADFB laser array module. Optical Fiber Communication Conference, Los Angeles, CA, USA. doi: 10.1364/OFC.2015.Tu3I.1. (Mar. 22–26, 2015).

[b10] Tsunoda Y. . 25.78-Gbps VCSEL-based optical transceiver with retime-embedded driver and receiver ICs. Optical Fiber Communication Conference, Los Angeles, CA, USA. doi: 10.1364/OFC.2015.Tu3G.4. (Mar. 22–26, 2015).

[b11] Nagashima K., Kise T., Ishikawa Y. & Nasu H. CDR-integrated Sn-Ag-Cu-solder reflow-capable miniature 28-Gbps × 4-channel optical modules. 2015 IEEE CPMT Symposium Japan (ICSJ), Kyoto, Japan. doi: 10.1109/ICSJ.2015.7357365. (Nov. 9–11, 2015).

[b12] Ghiasi A. Large data centers interconnect bottlenecks. Opt. Express 23, 2085–2090 (2015). 25836080 10.1364/OE.23.002085

[b13] Tatsumi T., Tanaka K., Sawada S., Fujita H. & Abe T. 1.3 μm, 56-Gbit/s EML module target to 400GbE. Optical Fiber Communication Conference, Los Angeles, CA, USA. doi: 10.1364/OFC.2012.OTh3F.4. (Mar. 4–8, 2012).

[b14] Kobayashi W. . Advantages of EADFB laser for 25 Gbaud/s 4-PAM (50 Gbit/s) modulation and 10 km single-mode fibre transmission. Electron. Lett. 50, 683–685 (2014).

[b15] Kanazawa S. . Compact flip-chip interconnection 8 × 50 Gbit/s EADFB laser array module for 400 Gbit/s transceiver. Electron. Lett. 50, 533–534 (2014).

[b16] Kanazawa S. . Compact flip-chip interconnection 112-Gbit/s EADFB laser array module with high eye-mask margin. IEEE/OSA J. Lightwave Technol. 32, 115–121 (2014).

[b17] IEEE P802.3ae 10Gbps Ethernet Task Force http://www.ieee802.org/3/ae/ (Last update: 2003, Jun. 12).

[b18] IEEE P802.3ba 40Gbps and 100Gbps Ethernet Task Force http://www.ieee802.org/3/ba/ (Last update: 2010, Jun. 19).

[b19] IEEE P802.3bm 40 Gbps and 100 Gbps Fiber Optic Task Force http://www.ieee802.org/3/bm/ (Last update: 2015, Jul. 22).

[b20] IEEE P802.3bs 200 Gbps and 400 Gbps Ethernet Task Force http://www.ieee802.org/3/bs/ (Last update: 2016, May 16).

[b21] Peters F. H. & MacDougal M. H.. High-speed high-temperature operation of vertical-cavity surface-emitting lasers. IEEE Photon. Technol. Lett. 13, 645–647 (2001).

[b22] Westbergh P., Gustavsson J. S. & Larsson A. high speed and high temperature operation of VCSELs. Optical Fiber Communication Conference, Los Angeles, CA, USA. doi: 10.1364/OFC.2015.M2D.5. (Mar. 22–26, 2015).

[b23] Chi K.-L. . Energy efficient 850 nm vertical-cavity surface-emitting lasers with extremely low driving-current density for >40 Gbit/sec error-free transmissions from RT to 85 °C. Optical Fiber Communication Conference, Los Angeles, CA, USA. doi: 10.1364/OFC.2015.M2D.6. (Mar. 22–26, 2015).

[b24] Westbergh P. . 40 Gbit/s error-free operation of oxide-confined 850 nm VCSEL. Electron. Lett. 46, 1014–1016 (2010).

[b25] Moser P. . Error-free 46 Gbit/s operation of oxide-confined 980 nm VCSELs at 85 °C. Electron. Lett. 50, 1369–1371 (2014).

[b26] Westbergh P. . High-speed oxide confined 850-nm VCSELs operating error-free at 40 Gbps up to 85 °C. IEEE Photon. Technol. Lett. 25, 768–771 (2013).

[b27] Kuchta D. M. . A 50 Gbps NRZ modulated 850 nm VCSEL transmitter operating error free to 90 °C. IEEE/OSA J. Lightwave Technol. 33, 802–810 (2015).

[b28] Westbergh P. . High-speed 850 nm VCSELs with 28 GHz modulation bandwidth operating error-free up to 44 Gbit/s. Electron. Lett. 48, 1145–1147 (2012).

[b29] Westbergh P. . High-speed 850 nm VCSELs operating error-free up to 57 Gbit/s. Electron. Lett. 49, 1021–1023 (2013).

[b30] Kuchta D. M. . 64Gbps transmission over 57m MMF using an NRZ modulated 850 nm VCSEL. Optical Fiber Communication Conference, San Francisco, California, USA. doi: 10.1364/OFC.2014.Th3C.2. (Mar. 9–13, 2014).

[b31] Szczerba K., Westbergh P., Karlsson M., Andrekson P. A. & Larsson A. 70 Gbps 4-PAM and 56 Gbps 8-PAM using an 850 nm VCSEL. IEEE/OSA J. Lightwave Technol. 33, 1395–1401 (2015).

[b32] Shi J.-W. . Single-Mode, High-speed, and high-power vertical-cavity surface-emitting lasers at 850 nm for short to medium reach (2 km) optical interconnects. IEEE/OSA J. Lightwave Technol. 31, 4037–4044 (2013).

[b33] Tatum J. A. The evolution of 850 nm VCSELs from 10 Gbps to 25 and 56 Gbps. Optical Fiber Communication Conference, San Francisco, California, USA. doi: 10.1364/OFC.2014.Th3C.1. (Mar. 9–13, 2014).

[b34] Shibata M. & Carusone A. C. A 26-Gbps 1.80-pJ/b CMOS-driven transmitter for 850-nm common-cathode VCSELs. Optical Fiber Communication Conference, Los Angeles, CA, USA. doi: 10.1364/OFC.2015.Tu3G.1. (Mar. 22–26, 2015).

[b35] Kuchta D. M. . A 71-Gbps NRZ modulated 850-nm VCSEL-based optical link. IEEE Photon. Technol. Lett. 27, 557–580 (2015).

[b36] Stepniak G. . 54 Gbps OOK transmission using single mode VCSEL up to 2.2 km MMF. Electron. Lett. 52, 633–635 (2016).

[b37] Li N. Y. . High-performance 850 nm VCSEL and photodetector arrays for 25 Gbps parallel optical interconnects. Optical Fiber Communication Conference, San Diego, CA, USA. doi: 10.1364/OFC.2010.OTuP2. (Mar. 21–25, 2010).

[b38] Kuchta D. M. . 120-Gbps VCSEL-based parallel-optical interconnect and custom 120-Gbps testing station,” IEEE/OSA J. Lightwave Technol. 22, 2200–2212 (2004).

[b39] Schow C. L. . 300 Gbps, 24-channel full-duplex, 850-nm, CMOS-based optical transceiver. Optical Fiber Communication Conference, San Diego, CA, USA. doi: 10.1109/OFC.2008.4528354. (Feb. 24–28, 2008).

[b40] Show C. L. . 225 Gbps Bi-directional integrated optical PCB link. National Fiber Optic Engineers Conference 2011, Los Angeles, CA, USA. doi: 10.1364/NFOEC.2011.PDPA2. (Mar. 6–10, 2011).

[b41] Tan M. R. . Low Cost, Injection molded 120 Gbps optical backplane. Optical Fiber Communication Conference, Los Angeles, CA, USA. doi: 10.1364/OFC.2011.PDPA4. (Mar. 6–10, 2011).

[b42] Westbergh P., Gustavsson J. S. & Larsson A. VCSEL arrays for multicore fiber interconnects with an aggregate capacity of 240 Gbit/s. IEEE Photon. Technol. Lett. 27, 296–299 (2015).

[b43] Ghiasi A. & Tang F. Enabling 850 nm VCSELs for 100GbE unretimed applications. Optical Fiber Communication Conference, Anaheim, CA, USA. doi: 10.1364/OFC.2013.OM2H.3. (Mar. 19–21, 2013).

[b44] Schow C. L. . A 24-Channel 300 Gbps 8.2 pJ/bit Full-Duplex Fiber-Coupled Optical Transceiver Module Based on a Single “Holey” CMOS IC. IEEE/OSA J. Lightwave Technol. 29, 542–553 (2011).

[b45] Nagashima K., Nishimura N., Izawa A., Kise T. & Nasu H. 28-Gbps × 24-channel CDR-integrated VCSEL-based transceiver module for high-density optical interconnects. Optical Fiber Communication Conference, Anaheim, California, USA. doi: 10.1364/OFC.2016.Th3G.3. (Mar. 20−22, 2016).

[b46] Tsunoda Y., . 24 to 34-Gbps × 4 multi-rate VCSEL-based optical transceiver with referenceless CDR. Optical Fiber Communication Conference, Anaheim, California, USA. doi: 10.1364/OFC.2016.Th4D.4. (Mar. 20–22, 2016).

[b47] Xie C. . 400-Gbps PDM-4PAM WDM system using a monolithic 2 × 4 VCSEL array and coherent detection. IEEE/OSA J. Lightwave Technol. 33, 670–677 (2015).

[b48] Tatum J. A. . VCSEL-based interconnects for current and future data centers. IEEE/OSA J. Lightwave Technol. 33, 727–732 (2015).

[b49] Alkharabsheh S. . A brief overview of recent developments in thermal management in data centers. ASME J. Electron. Package 137, 040801 (2015).

[b50] Arghode V. K. & Joshi Y. Experimental investigation of air flow through a perforated tile in a raised floor data center. ASME J. Electron. Package 137, 011011 (2015).

[b51] Chen G. A comparative study on the thermal characteristics of vertical‐cavity surface‐emitting lasers. J. Appl. Phys. 77, 4251–4258 (1995).

[b52] Liu Y., Ng W.-C., Choquette K. D. & Hess K. Numerical investigation of self-heating effects of oxide-confined vertical-cavity surface-emitting lasers. IEEE J. Quantum Electron. 41, 15–25 (2005).

[b53] Mutig A. . Temperature-dependent small-signal analysis of high-speed high-temperature stable 980-nm VCSELs. IEEE J. Sel. Top. Quantum Electron. 15, 679–686 (2009).

[b54] Lee Y. C. . Thermal management of VCSEL-based optoelectronic modules. Electronic 45th Components and Technology Conference, Las Vegas, Nevada, USA. doi: 10.1109/ECTC.1995.515309 (May 21–24, 1995).

[b55] Pu R., Wilmsen C. W., Geib K. M. & Choquette K. D. Thermal resistance of VCSELs bonded to integrated circuits. IEEE Photon. Technol. Lett. 11, 1554–1556 (1999).

[b56] Krishnamoorthy A. V. . 16 × 16 VCSEL array flip-chip bonded to CMOS VLSI circuit. IEEE Photon. Technol. Lett. 12, 1073–1075 (2000).

[b57] Choi J. H., Wang L., Bi H. & Chen R. T. Effects of thermal-via structures on thin-film VCSELs for fully embedded board-level optical interconnection system. IEEE J. Sel. Top. Quantum Electron. 12, 1060–1065 (2006).

[b58] Hino T., . A 10 Gbps × 12 channel pluggable optical transceiver for high-speed interconnections. 58th Electronic Components and Technology Conference. Orlando, Florida, USA. doi: 10.1109/ECTC.2008.4550231 (May 27–30, 2008).

[b59] Brusberg L., Schröder H. & Töpper M. Photonic system-in-package technologies using thin glass substrates. 11th Electronics Packaging Technology Conference. Singapore. doi: 10.1109/EPTC.2009.5416411 (Dec. 9–11, 2009).

[b60] Romero A. & Scott K. Cooling 8 × 100GbE switch blades with high power optical modules. 13th Thermal and Thermomechanical Phenomena in Electronic Systems. San Diego, California, USA. doi: 10.1109/ITHERM.2012.6231574 (May 30-Jun. 1, 2012).

[b61] Doany F. E. . Terabit/Sec VCSEL-based 48-channel optical module based on holey CMOS transceiver IC. IEEE/OSA J. Lightwave Technol. 31, 672–680 (2013).

[b62] Uemura T. . Thermal design of 28-Gb/s × 24-channel CDR-integrated VCSEL-based transceiver module. 2016 IEEE CPMT Symposium Japan. Tokyo, Japan. doi: 10.1109/ICSJ.2016.7801293 (Nov. 7–9, 2016).

